# *Novel cAMP binding protein-BP (CREBBP)* mutation in a girl with Rubinstein-Taybi syndrome, GH deficiency, Arnold Chiari malformation and pituitary hypoplasia

**DOI:** 10.1186/1471-2350-14-28

**Published:** 2013-02-23

**Authors:** Pierluigi Marzuillo, Anna Grandone, Ruggero Coppola, Domenico Cozzolino, Adalgisa Festa, Federica Messa, Caterina Luongo, Emanuele Miraglia del Giudice, Laura Perrone

**Affiliations:** 1 Department of Pediatrics “F. Fede”, Seconda Università degli Studi di Napoli, Via Luigi De Crecchio 2, 80138, Napoli, Italy; 2 Department of Internal Medicine, Seconda Università di Napoli, Via Sergio Pansini 5, 80131, Napoli, Italy

**Keywords:** Rubinstein-Taybi syndrome, GH deficiency, Arnold Chiari malformation, Syrinx, Pituitary hypoplasia

## Abstract

**Background:**

Rubinstein-Taybi syndrome (RTS) is a rare autosomal dominant disorder (prevalence 1:125,000) characterised by broad thumbs and halluces, facial dysmorphism, psychomotor development delay, skeletal defects, abnormalities in the posterior fossa and short stature. The known genetic causes are point mutations or deletions of the *cAMP-response element binding protein-BP (CREBBP)* (50-60% of the cases) and of the homologous gene *E1A-binding protein (EP300)* (5%).

**Case presentation:**

We describe, for the first time in literature, a RTS Caucasian girl, 14-year-old, with growth hormone (GH) deficiency, pituitary hypoplasia, Arnold Chiari malformation type 1, double syringomyelic cavity and a novel *CREBBP* mutation (c.3546insCC).

**Conclusion:**

We hypothesize that *CREBBP* mutation we have identified in this patient could be responsible also for RTS atypical features as GH deficiency and pituitary hypoplasia.

## Background

### Clinical overview

Rubinstein-Taybi syndrome (RTS) is an autosomal dominant disease that occurs in 1 out of 125,000 births. The first description of the syndrome by Rubinstein and Taybi was in 1963 [1].

RTS is characterized by broad and sometimes laterally deviated thumbs and halluces, downslanting palpebral fissures, apparent hypertelorism, long eyelashes, high-arched eyebrows, prominent nose with columella below the alae nasi, malpositioned ears with dysplastic helices, high-arched palate, hypoplastic maxilla. The facial expression is characteristic: showing grimacing or at least unusual smile. The finding of talon cusps at the permanent incisors can be helpful, as these are only rarely found in other entities [2].

Other physical findings may include a variety of congenital heart defects, joint hypermobility and skin anomalies (hirsutism, naevus flammeus on the forehead, and keloid formation) [3]. Mental retardation is usual with an average IQ between 35 and 50, but cognitive functioning outside these limits may occur [4].

Craniospinal and posterior fossa abnormalities have been commonly reported in association with RTS, including large foramen magnum, microcranium, spina bifida occulta, cervical hyperkyphosis, dens hypoplasia, cervical instability of C1-C2, cervical spondylolisthesis and scoliosis [5].

The association between RTS and Arnold-Chiari malformation has been described in four patients, complicated by syrinx in two of them [6-8].

Affected patients are also characterised by short stature with indirect measures of growth hormone usually found normal. There are no descriptions of growth hormone (GH) deficiency in this syndrome and therefore data on the need, effectiveness, or safety of growth hormone in children with this syndrome are not yet available in literature.

### Molecular and genetic basis

The first descriptions of cytogenetic anomaly in RTS were in 1991 [9-11]. The syndrome is autosomal dominant but reports of transmission are rare, the overwhelming majority of cases being caused by de novo mutations [12,13].

The known genetic causes are mutations of the genes *CREBBP* (OMIM 600140, *cAMP-response-element binding protein-BP*), located at chromosome 16p13.3 and *EP300* (OMIM 602700, *E1A-binding protein*), a *CREBBP* homolog, located at chromosome 22q13.2.

*CREBBP* mutations are found in 50-60% and EP300 mutations in 5% of RTS cases [14,15]. The *CREBBP* gene is involved in different signalling pathways and in certain cellular functions, such as DNA repair, cell growth, differentiation, apoptosis and tumor suppression.

The mutational spectrum is represented mainly from frameshift, nonsense, splice site and missense mutations. Less frequently large deletions (of one or more exons) and rarely balanced inversions and translocations have been found. Mutations may remove 5′ or the 3′ end of *CREBBP* and adjacent genomic segments, which causes the 16p13.3 contiguous gene deletion syndrome [16-18].

The genetic cause of RTS remains unknown in about 40% of the patients.

## Case presentation

We report a RTS girl born at gestional age of 38 weeks. Her birth weight and her length were 2,830 g and 48 cm respectively. Her newborn features included broad thumbs and toes, hypoplastic maxilla, malpositioned ears with dysplastic helices and hypertelorism.

Mutational analysis showed a frameshift mutation (c.3546insCC) of the *CREBBP* gene due to the insertion of two cytosines. This mutation, never described in literature, leads to a stop codon which probably causes nonsense mediated decay.

During childhood she showed growth and development delay. At the age of 11-year-old, when she came to our attention after being lost to follow-up, the stature was −3.5 standard deviation score (sds), the weight was between 10°-25° percentile, bone age, obtained by X-ray of non dominant hand and wrist and determined by TW2 method, was 9 years.

**Figure 1:**
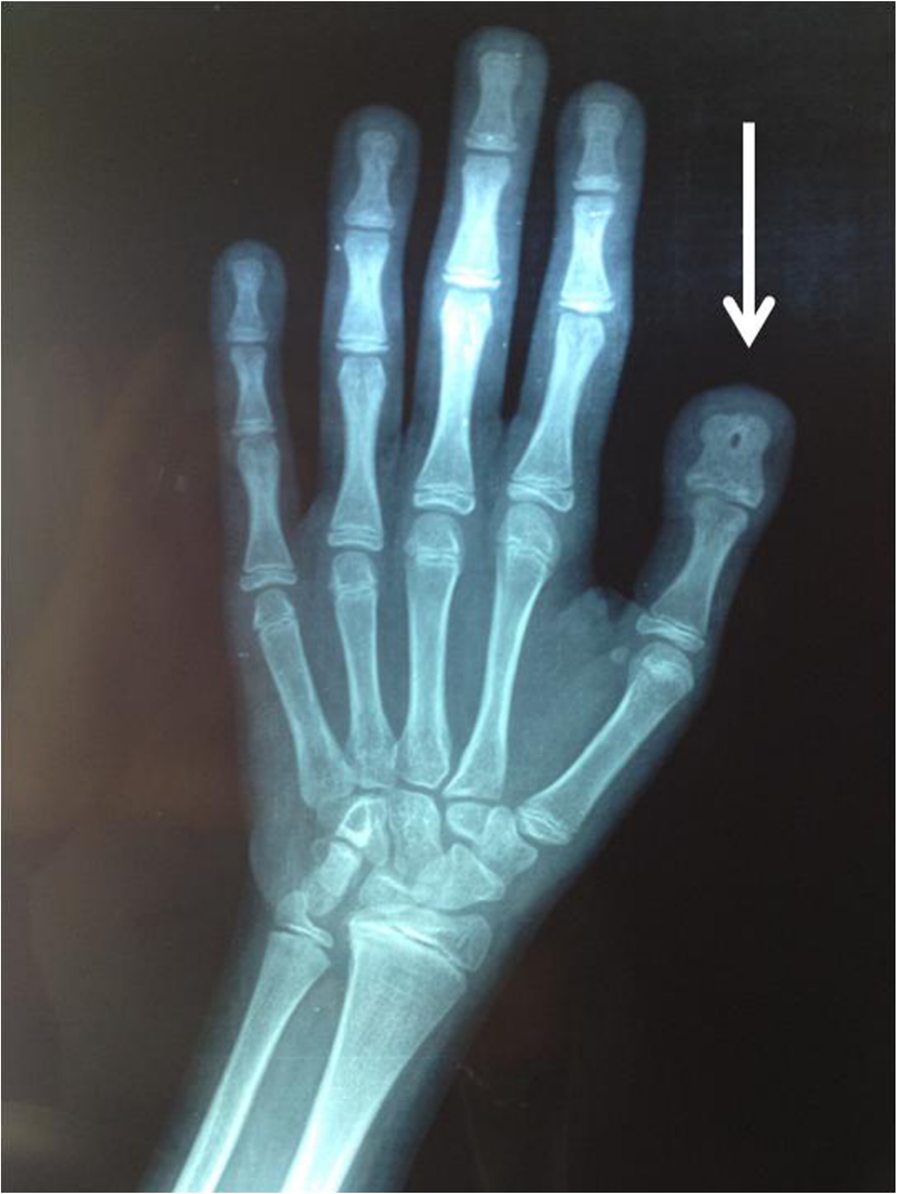
**This hand wrist radiograph was made when she was 14 years old.** The arrow shows a duplication of terminal phalanx of the thumb.

Pubertal Tanner stage was PH1 B1. She had scoliosis, mental retardation, microcephaly, long eyelashes, high-arched palate, beaked nose, duplication of terminal phalanx (Figure 1) of the thumbs.

Thyroid function was normal, cortisol levels at 8:00 a.m. and sexual hormones were normal for the age. Because of low IGF-1 (< 3° percentile), an arginine and then a clonidine provocative test for GH secretion were performed. GH peak levels were both < 7 ng/dL showing a GH deficiency. She started GH therapy at the dosage of 0.020 mg/kg/day with good improvement of growth velocity.

**Figure 2:**
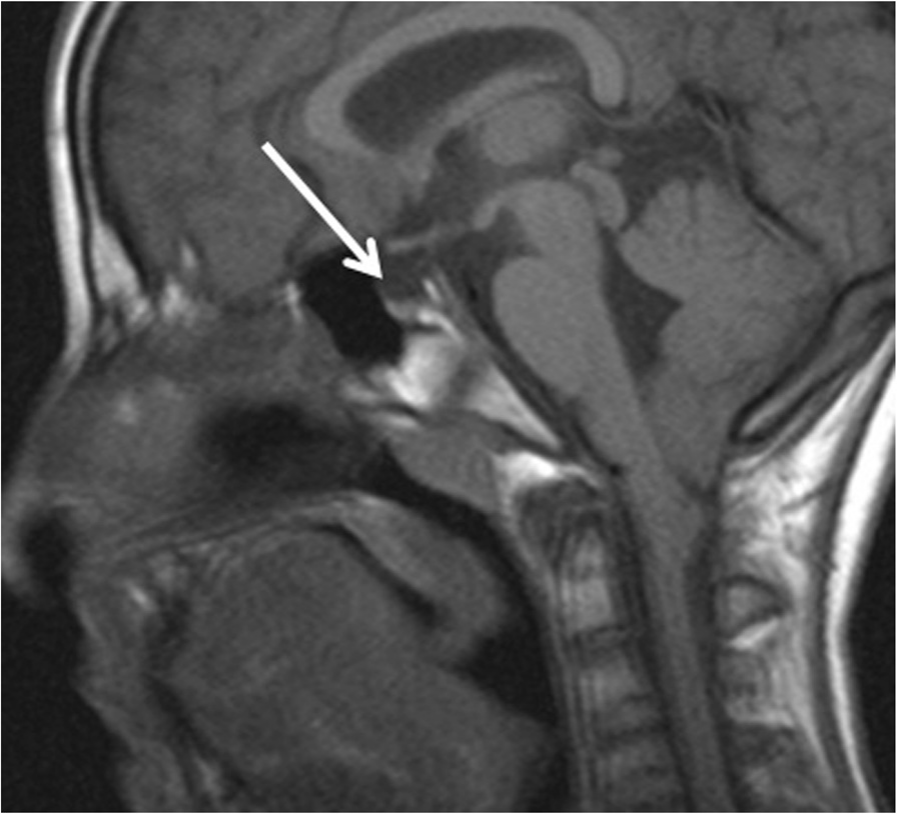
The arrow indicates the pituitary gland, which is hypoplastic.

**Figure 3:**
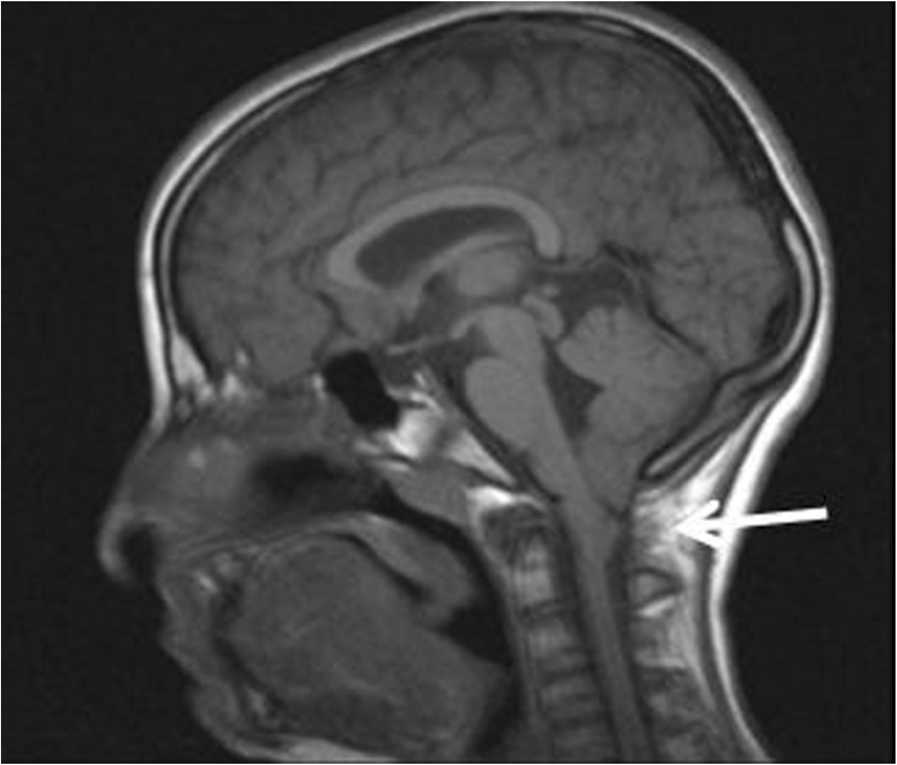
The arrow shows Arnold Chiari malformation type 1.

**Figure 4:**
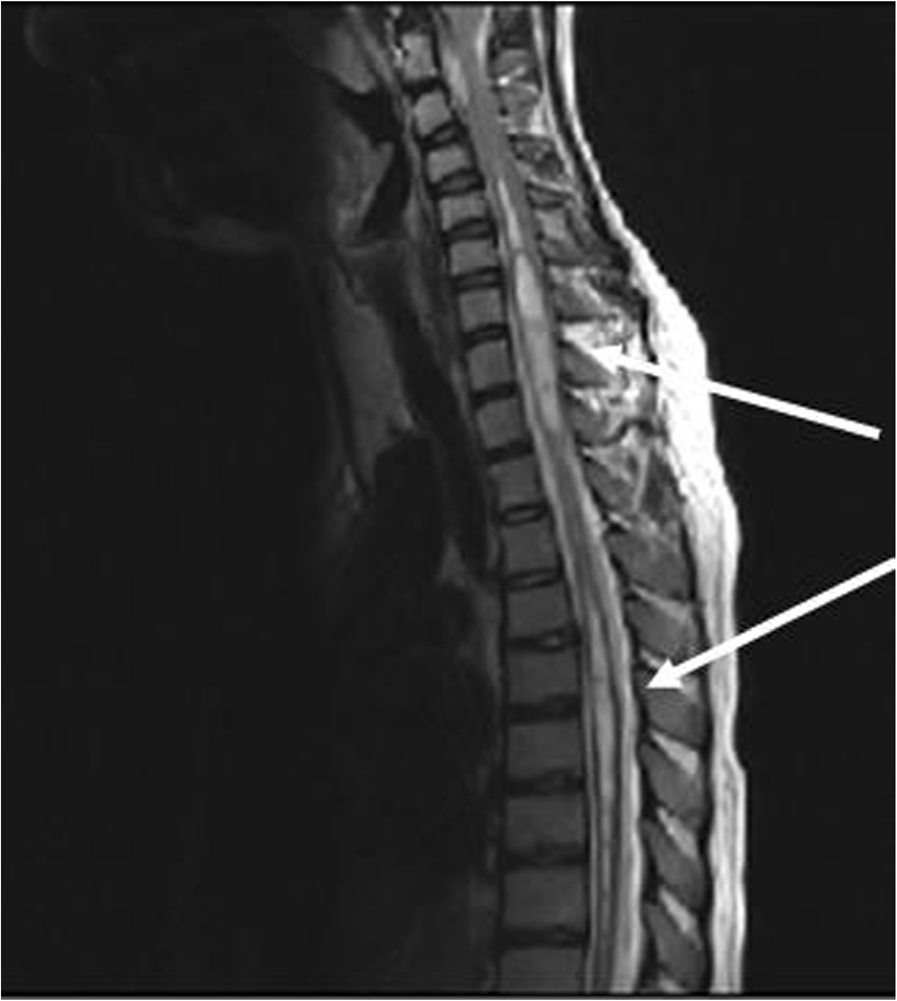
In this image, two syringomyelic cavity are evident, one of these is to cervical level, C5-T2 (thick arrow), and the other one to dorsal level, T5-T9 (thin arrow).

When she was 14-year-old, the stature was −2.7 sds and the weight between 3°-10° percentile. Bone age showed a delay of 1.5 years. Pubertal Tanner stage was PH2 B2. A brain magnetic resonance (MRI), performed as soon as the patient was cooperative enough not to require narcosis and parents gave their consent, showed pituitary hypoplasia (Figure 2) and Arnold Chiari malformation type1 (Figure 3). A medullary MRI scan showed a double syringomyelic cavity (Figure 4) requiring surgical treatment. Interestingly, we observed the complete absence of symptoms related to Arnold Chiari malformation and syrinx.

**Figure 5:**
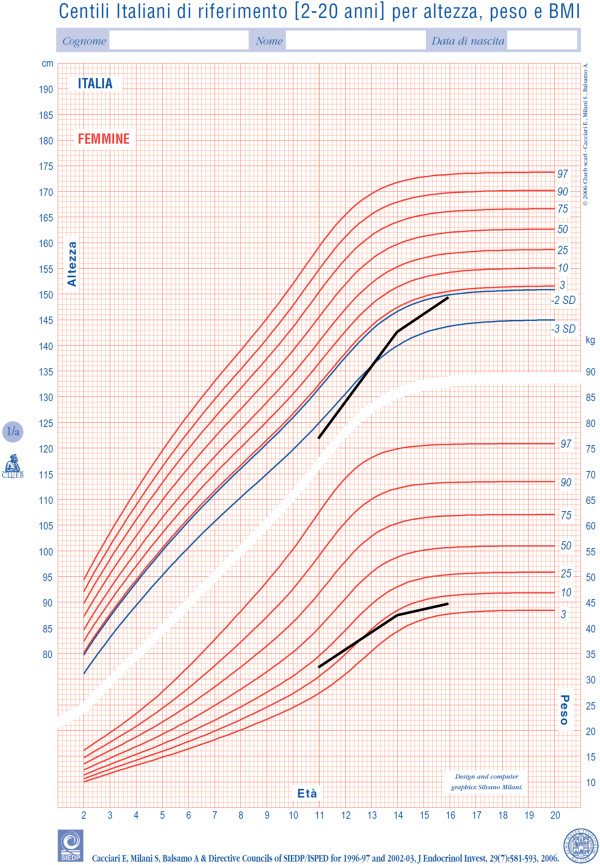
Growth of the patient during the GH therapy.

At 16-year-old, GH dose had been increased to 0.033 mg/Kg/day and the stature of the patient was −2.1 sds (Figure 5), the grade of scoliosis was constant. No side effects appeared during GH treatment.

## Discussion

The distinctive feature of the RTS girl we describe is the concurrent presence of GH deficiency, pituitary hypoplasia, Arnold Chiari malformation type 1 and double syringomyelic cavity. The presence of pituitary hypoplasia and GH deficiency in RTS patient, has never been reported so far.

Although frameshift and/or stop codons in *CREBBP* gene have been reported in literature, the mutation we have found is novel [6]. This case adds new clinical pieces to the syndromic puzzle of this rare syndrome.

### Arnold-Chiari malformation

RTS is associated with craniocervical and spinal cord complications. In literature patients with RTS affected by os odontoideum, C1-C2 instability, dens hypoplasia, scoliosis, cervical myelopathy, Chiari malformation, tethered spinal cord, large foramen magnum, vertebral fusion, microcranium, craniovertebral junction stenosis have been described [6,19,20].

The most serious consequences of Arnold Chiari malformation are attributable to syringomyelia formation with dysesthesias, paresthesias, anesthesias, incontinence, motor weakness, headache, abnormal reflexes as presenting signs [21]. However, our patient did not show any of these symptoms.

Little literature exists linking RTS to the occurrence of Chiari malformation. A case report examined a 2-year-old patient with RTS and a Chiari I malformation without syrinx who presented with a microdelection of 16p13.3 involving the *CREBBP* gene and additionally *ADCY9* and *SRL* genes. The authors of this article postulated that the Chiari I malformation may be related to a contiguous gene syndrome associating the haploinsufficiency of the neighboring genes to the etiology of Chiari malformation [8]. Another case report involving a 4-year-old Korean-Japanese RTS patient with a genetically confirmed *CREBBP* gene mutation with a C insertion in exon 31 discovered a Chiari I malformation. No syrinx was described in this patient’s imaging [7].

Finally, a recent case report described 13-year-old identical twin girls with RTS, Chiari malformation and extensive multioculated spinal cord syrinxes. The twins were both confirmed to possess an identical p.Arg2004 gene mutation in the *CREBBP* gene that is predicted to cause loss of normal protein function through premature protein truncation [6].

Since our patient also had a nonsense mutation in *CREBBP* leading to a premature stop codon, Chiari malformation and syrinx, this excludes the possibility of a contiguous gene syndrome (RTS + Chiari malformation + syrinx) previously postulated and confirms the single gene effect of *CREBBP* gene mutation.

### Growth hormone deficiency and pituitary hypoplasia

This is the first report of GH deficiency in a RTS patient. Indirect measures of growth hormone secretion have usually been reported normal in individuals with RTS in literature [20] but these patients have never been tested for growth hormone deficiency by dynamic testing.

Although the association between growth hormone deficiency and RTS could be coincidental, there are some possible pathogenic links between GH deficiency and RTS. *CREBBP* mutations could influence growth hormone secretion at multiple levels of the hypothalamic-pituitary-somatomedin axis, including both the hypothalamus and pituitary. A study using conditional *cAMP response element binding protein (CREB)* mutant mice showed that selective loss of the *CREB* transcription factor in all the brain except pituitary resulted in reduced postnatal growth consistent with dwarfism caused by GH deficiency and consequent reduction in IGF-1 mRNA expression in the liver. In addition the authors demonstrated that mice with *CREB* mutation exhibited pituitary hypoplasia. These findings show that *CREB* is required for the efficient function and development of pituitary probably having an effect at hypothalamic level, as *CREB* expression in pituitary in this animal model was normal [22,23]. Since *CREBBP* is an important *CREB* co-activator, the possibility exists that also *CREBBP* mutations at hypothalamic level can play a role in GH deficiency and pituitary hypoplasia. It has been demonstrated that, at pituitary level, *CREBBP* interacts as cofactor with Pit-1. Pit-1 is an important transcription factor that promotes growth hormone and stimulates somatic growth [24]. Therefore, an impairment of *CREBBP*/Pit-1 interaction could affect pituitary development and/or GH secrection.

## Conclusions

We propose that, since in this syndrome there is development delay with sometimes incapacity to describe symptoms, MRI imaging screening for Chiari malformation should be performed in all RTS patients at the first signs and/or suggestive symptoms (headache, neck pain, vertigo, sensory changes and ataxia or poor coordination) or, if asymptomatic, when patients are cooperative enough not to require narcosis (63% of patients with Chiari malformation can be asymptomatic) [25]. If patients have severe mental retardation, to strengthen the indication to perform an MRI, it could be useful, to use pain scales specific for children with severe cognitive impairment [26] to evaluate possible signs of discomfort related to Arnold-Chiari malformation.

To discriminate if there is a causal link between growth hormone deficiency and RTS, it could be interesting to study GH secretion in more patients with RTS to disclose if GH deficiency could play an causative role in short stature in RTS patients with *CREBBP* mutation.

Finally, the possibility exists that *CREBBP* gene mutation could be responsible both for GH deficiency and pituitary hypoplasia in our patient.

## Consent

Written informed consent was obtained from the patient’s guardian for publication of this case report and any accompanying images. A copy of the written consent is available for review by the Series Editor of this journal.

## Abbreviations

RTS: Rubinstein-Taybi syndrome;CREBBP: *cAMP-response element binding protein-BP*;EP300: *E1A-binding protein*;MRI: Magnetic resonance

## Competing interests

The authors declare that they have no competing interests.

## Authors’ contributions

PM drafted the manuscript. PM, EMDG and LP participated in the conception and the design of the study. AG and RC designed and interpreted the molecular evaluations. FM, AF conducted the molecular analyses. CL, AG and LP examined the patient, collected the data relevant to this case report and made the clinical diagnosis of the patient. LP supervised the design and execution of the study. DC contributed to the drafting of the text and undertook some clinical evaluations. All authors have read, revised and approved the final version of the manuscript.

## Pre-publication history

The pre-publication history for this paper can be accessed here:

http://www.biomedcentral.com/1471-2350/14/28/prepub
